# Three-Port Laparoscopic Cholecystectomy as a Safe and Feasible Alternative to the Conventional Four-Port Laparoscopic Cholecystectomy

**DOI:** 10.7759/cureus.52196

**Published:** 2024-01-13

**Authors:** Abhik Chatterjee, Ranjan Kumar, Ashok Chattoraj

**Affiliations:** 1 Surgery, Tata Main Hospital, Jamshedpur, IND; 2 General Surgery, Manipal Tata Medical College, Jamshedpur, IND

**Keywords:** hassan’s technique, calot’s triangle, visual analog scale (vas), pre-emptive analgesia, bile duct injury, critical view of safety, four-port cholecystectomy, three-port cholecystectomy

## Abstract

Aims

A prospective observational study was performed to assess the feasibility and safety of three-port laparoscopic cholecystectomy. Parameters comprising age, sex, number of cases in which intra-operative difficulty were encountered, and outcomes such as number of cases that required conversion to four-port laparoscopic cholecystectomy, postoperative pain on the visual analog scale (VAS), and postoperative hospital stay were assessed. We also documented difficult cases that were operated successfully with three ports, and the number of cases that needed conversion to four ports along with the reason for the conversion.

Material and methods

The patients were operated upon in the supine position in all cases. A pre-emptive analgesia with 1% lignocaine was administered in all cases prior to making the incision. The first port was 10-mm supraumbilical and inserted by the open technique. After insertion of the umbilical port, pneumoperitoneum was created by maintaining a maximum pressure of 12 mmHg and a flow rate of 8 L/minute. A camera head with a 30° telescope was introduced in the peritoneal cavity, and diagnostic laparoscopy was performed. A 10-mm subxiphoid port and a 5-mm subcostal port were placed under vision, with the latter placed more lateral and inferior to the conventional port position for better triangulation and ergonomics. The outcomes measured were operative time, the number of cases requiring a fourth port, postoperative pain (VAS), and postoperative hospital stay (number of days patients stayed in the hospital post-surgery until discharge). Data were collected using MS Excel, and an analysis was performed using SPSS Version 21.0.

Results

Data of 102 patients were analyzed prospectively. The mean age of the patients was 50.98 years, with an SD of 16.88, and the gender ratio was 73:29 (female: male). The mean operative time was 52.68 ± 20.84 minutes, with an SD of 20.84. Difficulty was encountered in 18.6% of cases in the form of pericholecystic adhesions, aberrant Calot’s anatomy, empyema or mucocele of the gallbladder, or bleeding from the liver bed or cystic artery stump. Postoperative pain was less in our study due to the reduced number of ports and the use of a pre-emptive analgesia, with a mean VAS score of 1.22 and an SD of 0.56. The mean postoperative hospital stay was 1.08 days, with an SD of 0.31. We needed to convert to a four-port procedure for safety in 2.9% cases. The operative time and postoperative hospital stay in our study were similar to those of other studies, but our average pain score was less due to the use of the pre-emptive analgesia. Only three cases required conversion to four ports, and 99 cases were successfully managed with three ports without compromising safety. No bile duct injury occurred in any of our 102 cases.

Conclusion

From this study, we conclude that three-port cholecystectomy is feasible, and it can be performed even in difficult cases without compromising safety. The surgical time is similar to that of four-port cholecystectomy, and the postoperative stay is shorter. The decreased number of ports and the pre-emptive analgesia reduced postoperative pain, cosmesis was better, and the incidence of bile duct injury did not increase. The procedure can also be converted to four-port cholecystectomy at any time if safety is compromised. Therefore, three-port cholecystectomy is a viable and safe option in the treatment of gallstone disease.

## Introduction

Since the first laparoscopic cholecystectomy was performed by Phillip Mouret in 1987, it has become the standard for treating cholelithiasis [1]. Conventional laparoscopic cholecystectomies are done with four ports (umbilical, epigastric, right hypochondrium, and right lumbar) [2], but with the evolution of technology, laparoscopic cholecystectomy has undergone a multitude of modifications, such as three-port laparoscopic cholecystectomy, the MiniLap system using 3-5 mm trocars [3-5], and single-incision laparoscopic surgery [6]. Some have advocated for omitting the fourth port in laparoscopic cholecystectomy with the aim of reducing invasiveness and surgical trauma to minimize pain and achieve better cosmesis [7]. Reducing the number of ports can also lead to better patient satisfaction, better cosmesis, and earlier discharge and return to normal activity [8].

Omission of the fourth port requires better instrument manipulation and hand-eye coordination for effective manipulation of the gallbladder and dissection of Calot’s triangle [9]. In three-port laparoscopic cholecystectomy, the fundal traction and the retraction of Hartmann’s pouch are performed simultaneously by the left-hand instrument, which may be difficult in cases of long overhanging and distended gallbladder, as well as enlarged left lobe of the liver, which tend to obscure the critical view of safety [10].

To determine the feasibility of three-port laparoscopic cholecystectomy without compromising safety, we conducted a prospective observational study of three-port laparoscopic cholecystectomies at our institute. We also documented challenging cases managed by three ports and the number of cases that required conversion from three to four ports. In addition, we analyzed patient outcomes in terms of postoperative pain (visual analog scale [VAS]) and postoperative stay (i.e., day of surgery to day of hospital discharge). Safety was defined as carrying out the procedure without any major bleeding, injury to viscera in the vicinity (i.e., stomach, duodenum, or colon), or injury to bile ducts. The addition of the fourth port was considered a failure of three-port laparoscopic cholecystectomy, and the reasons for conversion are also considered.

Although three-port cholecystectomy is documented as an alternative to conventional four-port cholecystectomy [11,12], there are limited data supporting its feasibility and, more importantly, its safety. In this study, we aim to demonstrate that three-port laparoscopic cholecystectomy is a feasible and safe substitute for the more commonly performed four-port laparoscopic cholecystectomy. Three-port laparoscopic cholecystectomy can be performed not only in routine cases but also in anticipated cases of difficult gallbladders, such as in cases of male sex, post-endoscopic retrograde cholangiopancreatography (post-ERCP) and stenting, post-pancreatitis, empyema of the gallbladder, and mucocele of the gallbladder, where dense adhesions of gallbladder with surrounding viscera may be encountered and achieving a critical view of safety might be difficult due to unclear anatomy.

## Materials and methods

Study design

This prospective study was based on data collected between December 2020 and February 2022 at Tata Main Hospital, Jamshedpur, Jharkhand, India. A total of 102 patients with symptomatic cholelithiasis diagnosed via abdominal ultrasound underwent elective three-port laparoscopic cholecystectomy. The inclusion criteria of the study were all patients 18 years of age or older with ultrasonographic evidence of cholelithiasis. The exclusion criteria were acute calculous cholecystitis (diagnosed clinically or radiologically) and cases that were converted to open cholecystectomy due to unclear anatomy, bleeding, dense adhesions, Mirizzi syndrome, or acute pancreatitis (diagnosed clinically according to laboratory parameters or radiologically). In cases of choledocholithiasis, the patient first underwent ERCP with common bile duct clearance ± stenting before being posted for elective three-port cholecystectomy. Patients with acute calculous cholecystitis and acute pancreatitis were managed conservatively and planned for interval cholecystectomy after six weeks.

Surgical technique

The patients were operated upon in the supine position in all cases using a Karl-Storz (Model 9627NB/KS-27, Karl-Storz, Tuttlingen, Germany) laparoscopic set. A pre-emptive analgesia with 1% lignocaine was administered in all cases prior to making the incision. The first port was 10-mm supraumbilical using the open (Hassan’s) technique [13]. After insertion of the umbilical port, pneumoperitoneum was created by maintaining a maximum pressure of 12 mmHg and a flow rate of 8 L/min. A camera head with a 30° telescope was introduced in the peritoneal cavity, and diagnostic laparoscopy was performed. A 10-mm subxiphoid port and a 5-mm subcostal port were placed under vision. The subcostal port was placed more lateral and inferior to the conventional port position for better triangulation and ergonomics. No suture was used to suspend the gallbladder from the abdominal wall. The gallbladder was retracted using long 5-mm grasping forceps through the subcostal port. Calot’s triangle was dissected using Maryland forceps, the cystic duct and cystic artery were identified, a critical view of safety [10] was established, and then the structures were clipped using a 10-mm clip applicator and divided using 5-mm endo-scissors. The gallbladder was dissected off the liver using a hook and monopolar cautery and retrieved through the umbilical port. All ports were removed under vision, and the pneumoperitoneum was deflated. The rectus sheath of the umbilical port was closed with Vicryl 2-0 suture, and the skin was approximated using skin staplers.

**Figure 1:**
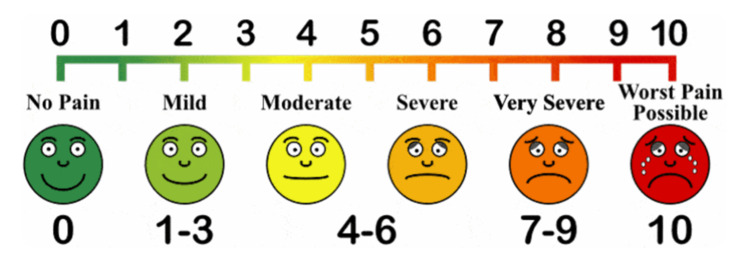
Visual analog scale

A single dose of acetaminophen injection (500 mg IV) was administered during the postoperative period; one hour after administration, postoperative pain was assessed using VAS (Figure 1). In cases in which the patient still experienced pain in the postoperative period, an additional dose of acetaminophen injection was administered on an SOS basis. The measured outcomes comprised operative time, number of cases that required insertion of the fourth port, postoperative pain (assessed using the VAS), and postoperative hospital stay (i.e., the number of days the patient stayed in the hospital post-surgery until discharge). All patients were allowed oral feeding six hours after surgery and discharged depending on oral diet acceptance and postoperative pain score. Patients were followed up on the 10th postoperative day for suture removal.

Statistical analysis

Data were collected using MS Excel (Microsoft Corp., Redmond, WA). Continuous variables were expressed as mean ± standard deviation and as range, and categorical variables were expressed as number and percentage. The analysis was conducted using SPSS Version 21.0 (IBM Corp., Armonk, NY).

## Results

In the study, data from 102 patients were evaluated. out of 102 patients, 99 underwent a three-port laparoscopic cholecystectomy. A four-port laparoscopic cholecystectomy was performed in three patients.

Patient characteristics

The mean age of the patients was 50.98, with an SD of 16.88 (Table 1), and the gender ratio of the patients was 73:29 (female:male).

**Table 1:** Age and gender distribution

Variables (n=102)	N (%)
Age	
≤30 years	15 (14.7)
31-50 years	31 (30.4)
51-70 years	42 (41.2)
>70 years	14 (13.7)
Gender	
Female	73 (71.6)
Male	29 (28.4)

Surgical parameters

The mean operating duration was 52.8 minutes, with an SD of 20.84. The range was 100 minutes, with a minimum time of 15 minutes and a maximum time of 115 minutes (Table 2). Technical difficulty was encountered in 19 out of 102 cases (Table 2). Three of these cases had choledocholithiasis, for which they had to undergo ERCP prior to cholecystectomy, two cases had empyema of the gallbladder, three cases had dense pericholecystic adhesions, three cases had aberrant Calot’s anatomy, two cases had mucocele of the gallbladder, and two cases had a prior history of pancreatitis due to gallstones. There was bleeding from the gallbladder bed in three cases, and one case had bleeding from the cystic artery stump. The cases in which we encountered mucocele and empyema had thick and edematous gallbladder walls, with adhesions to the omentum, the colon, and the duodenum. Adhesiolysis and hemostasis were performed in all of the above-mentioned cases using three ports, and there was no injury to the surrounding viscera. In addition, no bile duct injury occurred in any of the cases managed by three ports.

**Table 2:** Surgical and postoperative parameters

Name of Variables (n=102)	Mean (SD)	Range (Min, Max)
Age	50.98 (16.88)	65 (15, 80)
Pain score	1.22 (0.56)	3 (0, 3)
Operation time (in minutes)	52.68 (20.84)	100 (15, 115)
Postoperative stay	1.08 (0.31)	2 (1, 3)
Name of Variables (n=102)	Name of Grouping Variables	N (%)
Pain score	Score 0	6 (5.9)
	Score 1	69 (67.6)
	Score 2	26 (25.5)
	Score 3	1 (1.0)
Difficulty	Yes	19 (18.6)
	No	83 (81.4)
Conversion	Yes	83 (81.4)
	No	99 (97.1)
Postoperative stay	Day 1	95 (93.1)
	Day 2	6 (5.9)
	Day 3	1 (1.0)

In three out of 102 cases (Table 2), we had to use a fourth port as a result of compromised safety due to different reasons: dense adhesions in Calot’s triangle were seen in two cases, where we could not delineate the anatomy successfully using three ports; in one case, the clip on the cystic artery slipped. Therefore, we used a fourth port to retract the gallbladder and take control of the cystic artery, which was re-clipped to prevent any inadvertent thermal or mechanical injury to the bile ducts and their vascularity.

Postoperative parameters

The mean postoperative pain score was 1.22, with an SD of 0.56. VAS scores of 0, 1, 2, and 3 were seen in six, 69, 26, and one patient(s), respectively. The mean postoperative hospital stay was 1.08 days, with an SD of 0.31. Also, 95 patients were discharged on postoperative day 1, six patients were discharged on postoperative day 2, and one patient was discharged on postoperative day 3 (Table 2).

## Discussion

Since its inception, laparoscopic cholecystectomy has undergone many modifications, including three-port laparoscopic cholecystectomy. In this study, data on 102 patients were analyzed prospectively. In 18.6% of cases, difficulty was encountered in the form of pericholecystic adhesions, aberrant Calot’s anatomy, empyema or mucocele of the gallbladder, or bleeding from the liver bed or cystic artery stump. Postoperative pain was less when compared to other similar studies due to the reduced number of ports and the use of pre-emptive analgesia, with a mean VAS score of 1.22 and an SD of 0.56, and a mean postoperative hospital stay of 1.08 days and an SD of 0.31. In 2.9% of cases, we needed to convert to four-port laparoscopic cholecystectomy to complete the procedure safely. The operative time and postoperative hospital stay in our study were similar to those of other studies, but the pain score in our study was lower due to the use of the pre-emptive analgesia (Table 3).

**Table 3:** Data from other studies VAS, visual analog scale

Study	Age	Female/male	Operative time (minutes)	VAS score	Hospital stay (days)
Agrusa et al. [7]	46 (18-62)	40/11	57. 5	-	-
Kumar et al. [9]	38.22±13.67	30/6	47.3± 29.8	2.19 ±1.06	1.19 ± 0.06
Omar et al. [14]	41 ± 10	58/41	45.2 ±11.8	3 ±1.5	1.2 ± 0.9
Cheng et al. [15]	42.3 ± 11.0	191/124	33.5 ± 9.0	2.3 ± 1.3	1.4 ± 0.7
Jung et al. [16]	45.6 ± 13.8	14/16	34.9 ± 5.75	-	3.7 ± 0.8
Pan et al. [17]	45.2 ± 11.0	31/22	38.5 ± 22.0	3.5 ± 1.6	1.0 ± 0.2
Khorgami et al. [18]	41.7 ± 11.2	20/10	54.2 ± 14.4	5.0±1.4	-

In this study, we tried to establish that three-port laparoscopic cholecystectomy is a safe and feasible alternative to conventional four-port laparoscopic cholecystectomy, although there is limited literature to suggest the same. Only three cases required conversion to four ports, and 99 cases were successfully managed with three ports without compromising safety. No bile duct injury occurred in any of our 102 cases. Not only did we manage uncomplicated gallbladders with the three-port technique, but also the so-called difficult gallbladders (i.e., male sex, post-ERCP and stenting, post-pancreatitis, and empyema and mucocele of the gallbladder) were also managed safely without any postoperative complication or bile duct injury.

The difficulty that we encountered in our cases was when the gallbladder was long or when the left lobe of the liver was enlarged, which, if not retracted properly, kept falling forward and obscuring Calot’s triangle. This difficulty was overcome by gaining experience as we performed more cases with proper traction and counter-traction using the laparoscopic instruments. Such retraction was relatively easy in difficult cases with adhesions and inflamed gallbladders, as they did not fall forward due to the adhesions and inflammation. The use of the pre-emptive analgesia helped us achieve low pain scores, which supported early recovery and early return to daily activities.

This study was limited in that we did not have a control group for comparison. However, we were able to compare our data with those of other studies (Table 3), and we can say that three-port laparoscopic cholecystectomy can be mastered using proper technique and supervision and that it is a safe alternative to four-port cholecystectomy. Furthermore, difficult gallbladders can also be managed safely with the three-port technique.

Some concerns exist, such as a reduced number of ports can lead to a higher incidence of bile duct injuries [19]. However, bile duct injuries can be avoided by establishing a critical view of safety in all cases. The traction that we apply on the gallbladder should be superolateral rather than completely lateral, which will avoid excessive tenting of the common bile duct and subsequent bile duct injury. Excessive lateral traction may also avulse the duct or artery, and therefore one should be careful not to apply too much traction. Other studies conducted in the past have confirmed the safety of the procedure [12,20-21]. Our study was a prospective observational study with a sample size of 102, but to further establish the safety and feasibility of three-port laparoscopic cholecystectomy, a comparative study with a larger sample size is required.

## Conclusions

From the results of this study, we conclude that three-port laparoscopic cholecystectomy is feasible and can even be performed in difficult cases without compromising safety. The surgical time is similar to that of four-port cholecystectomy, and the postoperative stay is shorter. The decreased number of ports along with a pre-emptive analgesic reduces postoperative pain, cosmesis is better, and the incidence of bile duct injury does not increase. Having said this, the procedure can be converted to four-port laparoscopic cholecystectomy at any time if safety is compromised. Hence, three-port laparoscopic cholecystectomy is a viable and safe option in the treatment of gallstone disease.
